# Age-dependent phenotypes of cognitive impairment as sequelae of SARS-CoV-2 infection

**DOI:** 10.3389/fnagi.2024.1432357

**Published:** 2025-01-07

**Authors:** Gabriela Gonzalez Aleman, George D. Vavougios, Carmela Tartaglia, Nalakath A. Uvais, Alla Guekht, Akram A. Hosseini, Vincenzina Lo Re, Catterina Ferreccio, Giovanni D'Avossa, Hernan P. Zamponi, Mariana Figueredo Aguiar, Agustin Yecora, Mohammad Zia Ul Haq Katshu, Vasileios T. Stavrou, Stylianos Boutlas, Konstantinos I. Gourgoulianis, Camila Botero, Francisco González Insúa, Santiago Perez-Lloret, Mikhail Zinchuk, Anna Gersamija, Sofya Popova, Yulia Bryzgalova, Ekaterina Sviatskaya, Giovanna Russelli, Federica Avorio, Sophia Wang, Paul Edison, Yoshiki Niimi, Hamid R. Sohrabi, Elizabeta B. Mukaetova Ladinska, Daria Neidre, Gabriel A. de Erausquin

**Affiliations:** ^1^Department of Psychology, School of Psychology and Psychopedagogy, Universidad Catolica Argentina, Buenos Aires, Argentina; ^2^Department of Neurology, Medical School, University of Cyprus, Nicosia, Cyprus; ^3^Department of Respiratory Medicine, University of Thessaly, Larissa, Greece; ^4^Tanz Centre for Research in Neurodegenerative Diseases, University of Toronto, Toronto, ON, Canada; ^5^Memory Clinic, Department of Neurology, Toronto Western Hospital, Toronto, ON, Canada; ^6^Department of Psychiatry, Iqraa International Hospital and Research Centre, Calicut, India; ^7^Department of Neurology, Moscow Research and Clinical Centre for Neuropsychiatry, Moscow, Russia; ^8^Department of Neurology, Pirogov Russian National Research Medical University, Moscow, Russia; ^9^Nottingham University Hospitals NHS Trust, Nottingham, United Kingdom; ^10^Nottingham Sir Peter Mansfield Imaging Centre, University of Nottingham, Nottingham, United Kingdom; ^11^Neurology Service, Department of Diagnostic and Therapeutic Services, IRCCS ISMETT, Palermo, Italy; ^12^Department of Experimental Medicine and Clinical Neuroscience, University of Pittsburgh Medical Center (UPMC), Palermo, Italy; ^13^Department of Public Health School of Medicine, Pontificia Universidad Catolica de Chile, Santiago, Chile; ^14^Advanced Center for Chronic Diseases, ACCDiS, Santiago, Chile; ^15^School of Psychology and Sports Sciences, Bangor University, Bangor, United Kingdom; ^16^Secretariat for Mental Health and Addictions, Ministry of Health, Government of Jujuy, San Salvador de Jujuy, Argentina; ^17^Instituto San Lazaro de Neurociencias, Fundacion de Lucha contra los Trastornos Neurologicos y Psiquiatricos en Minorias, FULTRA, San Salvador de Jujuy, Argentina; ^18^Institute of Mental Health, University of Nottingham, Nottinghamshire Healthcare NHS Foundation Trust, Nottingham, United Kingdom; ^19^Health Observatory, Vice Rectorate for Research, Universidad Catolica Argentina, Buenos Aires, Argentina; ^20^Department of Psychiatry, Indiana University School of Medicine, Indianapolis, IN, United States; ^21^Indiana Alzheimer's Disease Research Center, Indianapolis, IN, United States; ^22^Department of Brain Sciences, Faculty of Medicine, Imperial College London, London, United Kingdom; ^23^Cardiff University, Cardiff, United Kingdom; ^24^Faculty of Medicine, University of Tokyo, Tokyo, Japan; ^25^Murdoch University Centre for Healthy Ageing, School of Psychology, Murdoch University, Murdoch, WA, Australia; ^26^Department of Psychology and Visual Sciences, University of Leicester, Leicester, United Kingdom; ^27^The Evington Centre, Leicester General Hospital, Leicester, United Kingdom; ^28^Laboratory for Brain Development, Modulation and Repair, Glenn Biggs Institute for Alzheimer's and Neurodegenerative Diseases, University of Texas Health San Antonio, San Antonio, TX, United States; ^29^Laboratory of Electrophysiology Imaging, Radiology Research Institute, University of Texas Health San Antonio, San Antonio, TX, United States; ^30^Department of Neurology, Joe & Teresa Long School of Medicine, University of Texas Health San Antonio, San Antonio, TX, United States

**Keywords:** COVID-19, cognitive decline, international cohort, age-dependent, long COVID

## Abstract

Cognitive changes associated with PASC may not be uniform across populations. We conducted individual-level pooled analyses and meta-analyses of cognitive assessments from eight prospective cohorts, comprising 2,105 patients and 1,432 controls from Argentina, Canada, Chile, Greece, India, Italy, Russia, and the UK. The meta-analysis found no differences by country of origin. The profile and severity of cognitive impairment varied by age, with mild attentional impairment observed in young and middle-aged adults, but memory, language, and executive function impairment in older adults. The risk of moderate to severe impairment doubled in older adults. Moderately severe or severe impairment was significantly associated with infection diagnoses (chi-square = 26.57, *p* ≤ 0.0001) and the severity of anosmia (chi-square = 31.81, *p* ≤ 0.0001). We found distinct age-related phenotypes of cognitive impairment in patients recovering from COVID-19. We identified the severity of acute illness and the presence of olfactory dysfunction as the primary predictors of dementia-like impairment in older adults.

## Introduction

COVID-19 is a disease caused by the coronavirus SARS-CoV-2. SARS-CoV-2 infection can cause severe respiratory disease, resulting in pneumonia and death. COVID-19 has now spread to 222 countries and territories worldwide, with more than 704 million cases and >7 million deaths [worldometers.info/coronavirus]. Since its emergence, our understanding of COVID-19 has evolved to recognize its systemic features and potentially long-lasting consequences. As the exposure of the population to SARS-CoV-2 accelerated, these symptoms became increasingly recognized as a syndrome known as post-acute sequelae of COVID-19 (PASC) or long COVID (Nalbandian et al., 2021; Proal and VanElzakker, 2021).

Epidemiological studies suggest that PASC continues to have a global impact. In 2021, a systematic review of studies involving >250,000 survivors of COVID-19 found that PASC was evident in more than half, and PASC symptoms that persisted for 6 months or longer after the acute phase of infection affected multiple organs, leading to respiratory, cardiovascular and neurological symptoms (Groff et al., 2021). In an analysis of nearly 1.5 million COVID-19 patients followed up for 2 years after the initial infection, the risk for cognitive impairment remained higher in the group exposed to COVID-19 (Taquet et al., 2022). In 2022, the Human Phenotype Ontology meta-analyzed findings from 81 PASC cohorts, indicating that cognitive impairment and other neuropsychiatric symptoms were consistent symptoms in PASC (Deer et al., 2021).

Neuropsychiatric PASC may be distinct from other multi-systemic manifestations. This may be explained by a biological interplay between SARS-CoV-2 infection and neurodegeneration (de Erausquin et al., 2021; Li et al., 2022). For instance, levels of neurodegenerative biomarkers such as total tau, p-tau181, glial fibrillary acidic protein (GFAP), and neurofilament light (NfL) were increased following COVID-19 infection in patients without a history of neurodegenerative disease (Frontera et al., 2022). Notably, plasma NfL, a marker of axonal damage, was high after SARS-CoV-2 infection in the absence of neurological manifestations, (Verde et al., 2022) but in association with increased levels of inflammatory cytokines (Hirzel et al., 2022). An interpretation of these findings suggests that SARS-CoV-2 may damage the central nervous system either directly (Meinhardt et al., 2021), or mediated by an abnormal immune response (Jarius et al., 2022). Regardless of the underlying mechanism, data from the UK Biobank show brain volume reductions in limbic and olfactory pathways (Douaud et al., 2022), placing anosmia and cognitive deficits within the same syndromic spectrum (Deer et al., 2021; de Erausquin et al., 2021; Zamponi et al., 2021; Galderisi et al., 2024). Taken together, these associations between clinical phenotypes, imaging, and underlying biology indicate that neurocognitive disorders may be a distinct phenomenon among other PASC manifestations (de Erausquin et al., 2021; Frontera and Simon, 2022).

The impact of the pandemic has not been uniform across human groups or regions of the world. Notably, and beyond obvious comorbidities that increase the risk of various diseases, such as obesity or diabetes mellitus (Lee et al., 2024), susceptibility to the infection's deleterious effects has been higher across groups from specific ancestries. In the U.S.A., the highest morbidity and mortality rates were found in Amerindian minorities, followed by African Americans and Hispanics (Lundberg et al., 2023). Similarly, Amerindian (Maya) ancestry was associated with Southern Mexico's highest morbidity and mortality rates (Rangel-Méndez et al., 2023). In the U.K., the highest morbidity and mortality rates were reported for Bangladeshi and Pakistani minorities, followed by Black Africans and Black Caribbean [Office for National Statistics (ONS), 2023] Notably, these differential effects have disappeared after the Omicron variant of SARS-CoV-2 became prevalent [Office for National Statistics (ONS), 2023]. Therefore, substantial human host factors influence SARS-CoV-2 infection, and several recent studies point to genetics as the primary contributor (Nakanishi et al., 2021; Garg et al., 2024). A genetic predisposition may be causally linked to some (but not all) PASC symptoms (Shenoy et al., 2023; Tang et al., 2023).

Genetically based protein alterations can lead to large differences in differential response to SARS-CoV-2 infection, and some manifestations of PASC may be influenced by genetic variants shared with other diseases. For cognitive decline, the APOE gene has long been implicated in the risk of Alzheimer's disease, and the e4/e4 genotype has been shown to increase susceptibility to COVID-19, excess neuroinflammation, and reduced antiviral response (Kuo et al., 2020; Farrer et al., 1997; Severe Covid-19 GWAS Group et al., 2020). Thus, APOE may play a causal role in the severity of neurological symptoms in infection. SARS-CoV-2 infection significantly alters molecular pathways implicated in brain inflammation, and certain viral entry factors are highly expressed in cells in the blood-brain barrier. Indeed, PASC cognitive sequelae may resemble early Alzheimer's disease, and olfactory dysfunction may be an accurate predictor of the severity of cognitive sequelae (Gonzalez-Aleman et al., 2022).

In sum, cognitive changes associated with PASC may not be uniform across populations, and refining clinical phenotypes is essential to pursuing biomarkers of genetic studies in the future. We carried out individual-level pooled data analyses from Argentina, Canada, Chile, Greece, India, Italy, Russia, and the United Kingdom (UK), harmonizing following published protocols (de Erausquin et al., 2022) to define cognitive impairment profiles.

## Results

### Description of the overall sample

The whole sample consisted of 3,537 (2,105 infected and 1,432 uninfected controls, Table 1). Participants ranged in age from 18 to 97 years. The mean duration of formal education was 10.66 ± 4.5 years, and the mean age was 52.81 ± 15.76 years; 36.5% were men and 64.5% were women.

**Table 1:** Description of cohorts participants.

	**N**	**Controls**	**Patients**	**Age**	**Education (in years)**
Argentina	866	137	729	67.13 ± 5.7	10.23 ± 4.72
Canada	97	0	97	52.23 ± 11.16	14.68 ± 3.62
Chile	467	361	106	57.1 ± 8.81	
Greece	252	47	205	54 ± 11.66	
India	340	169	171	37.29 ± 14.41	11.65 ± 3.61
Italy	28	6	22	57.23 ± 10.96	10.64 ± 4.07
Russia	1,416	689	727	47.63 ± 14.65	
UK	71	23	48	58.63 ± 11.49	
	3,537	1,432	2,105		

### Pooled analysis of individual-level data

**Figure 1:**
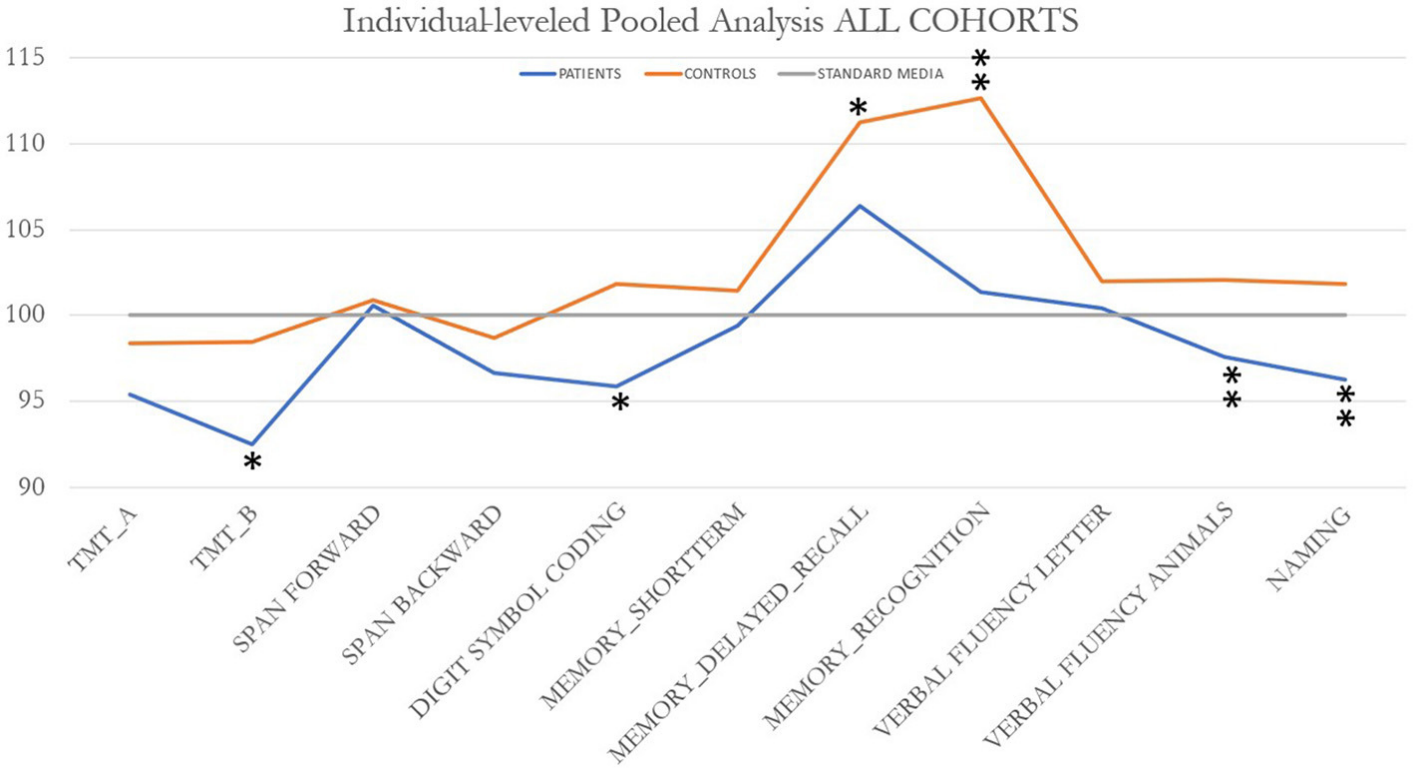
Profiles of cognitive impairment in PASC patients across all cohorts. Significant differences were found on Trail Making Test (Part B) (*p = 0.012); Symbol Digit Coding (Weschler intelligence battery; **p ≤ 0.0001); Memory delayed recall (**p = 0.009); recognition (**p < 0.0001), verbal semantic fluency (animals) (**p ≤ 0.0001); and naming (**p < 0.0001).

**Figure 2:**
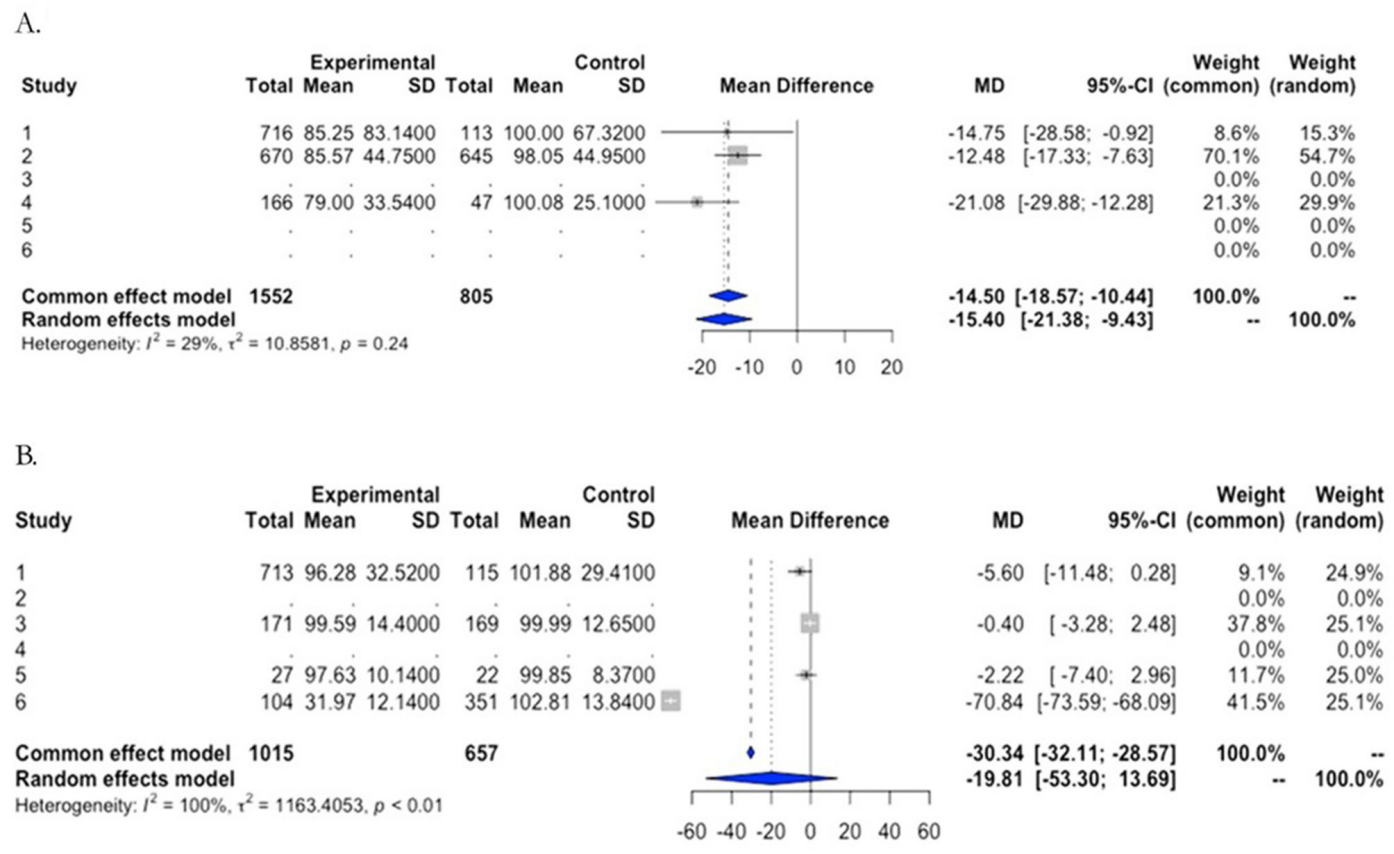
Meta-analyses of cognitive impairment by cohort. Forest plots of comparison of Trail Making Test B **(A)** and, as an example of a negative result, the Naming test **(B)**.

The cognitive performance of pooled, individual-level data was compared first task-wise for the entire sample. The infection performed significantly worse than the controls on the Trail Making Test (Part B), Symbol Digit Coding, Memory Delayed Recall, Recognition, Verbal Semantic Fluency (animals), and Naming (Figure 1). By contrast, a meta-analysis to test for differences between patients and controls by country of origin found that all patients showed impairment in executive function as indicated by performance only on the Trail Making Test-part B (Figure 2), suggesting loss of cognitive flexibility.

**Figure 3:**
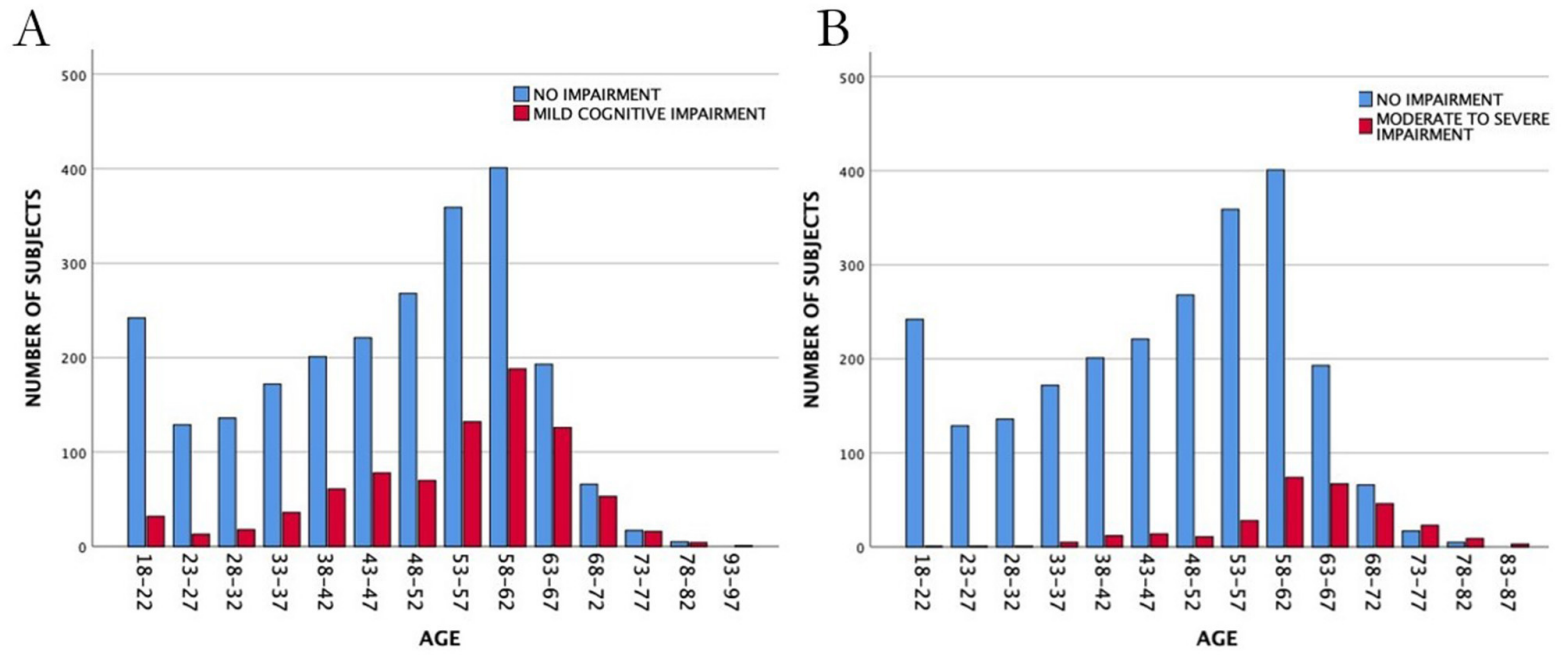
Frequency distributions for individuals with mild **(A)** or moderate to severe **(B)** cognitive impairment by age, regardless of diagnosis and across all cohorts. Whereas mild cognitive impairment follows a normal distribution largely overlapping (and not significantly different) from normal cognition, moderate to severe cognitive impairment is skewed toward older age and significantly different from the rest of the population (see main text).

Based on visual inspection of frequency plots by age, a bimodal distribution of overall impairment was identified, and an empirical cut-off point was identified at 59 years of age (Figure 3). Specifically, the distribution of mild cognitive impairment (1 domain only) by age was examined, along with the distribution of moderate to severe cognitive impairment (2 or 3 domains) (Figure 3). We tested the hypothesis of independence of these distributions and found it to be highly significant (t = −10.95, df = 642.86, *p* ≤ 0.0001). The median age in the mild cognitive decline group was 57.6, and in the moderate to severe cognitive impairment group, it was 65.8 years. Therefore, subjects 60 years of age or older (older adults) and subjects younger than 59 years of age (middle-aged and young adults) were treated as independent samples henceforth. The results are shown in Tables 2, 3.

**Table 2:** Pooled data all cohorts.

	**N**	**Mean ±**	**SD**	**ΔX**	**SE**	**t**	**df**	***p*-value**
**Trail-making test–part A**				4.26	3.53	1.2	1225	0.2300
Patients	933	111.09 ±	56.72					
Controls	294	106.83 ±	52.81					
**Trail-making test–part B**				−1.82	4.14	−0.44	624.58	0.6600
Patients	915	111.48 ±	56.71					
Controls	288	113.3 ±	75.90					
**Span forward**				−6.39	1.41	−4.52	605.985	**0.0010**
Patients	915	99.73 ±	24.46					
Controls	296	93.34 ±	19.93					
**Span backward**				−1.12	1.75	−0.64	1302	0.5200
Patients	1004	93.43 ±	26.51					
Controls	300	92.3 ±	27.04					
**Symbol digit coding**				1.21	2.60	0.45	704.542	0.6400
Patients	895	91.12 ±	50.74					
Controls	284	92.34 ±	34.31					
**Short-term memory**				−0.65	1.80	−0.35	534.964	0.7200
Patients	901	96.82 ±	30.94					
Controls	276	96.16 ±	25.99					
**Delayed recall memory**				10.26	2.85	3.6	1,424	**0.0010**
Patients	1024	96.89 ±	47.99					
Controls	402	107.15 ±	48.75					
**Recognition**				10.78	1.68	6.94	298,960	**0.0010**
Patients	779	98.62 ±	14.12					
Controls	249	109.4 ±	25.28					
**Verbal fluency-letter**				3.9	3.17	1.23	571	0.2100
Patients	255	88.9 ±	39.97					
Controls	318	92.81±	35.82					
**Verbal fluency-animals**				3.94	1.72	2.28	773.266	**0.0230**
Patients	1058	93.04 ±	28.05					
Controls	458	97 ±	32.07					
**Naming**				3.65	1.84	1.97	620.871	0.0500
Patients	781	95.33±	36.37					
Controls	264	98.98 ±	23.42					

Cognitive performance in older adults (60 years old and above) vs. non-infected controls. Bold lettering indicates statistical significance.

**Table 3:** Pooled data all cohorts.

	**N**	**Mean ±**	**SD**	**ΔX**	**SE**	**t**	**df**	***p*-value**
**Trail-Making Test- part A**				−0.33	2.59	−0.13	1057	0.89
Patients	556	91.27 ±	41.11					
Controls	503	90.49 ±	43.42					
**Trail-Making Test- part B**				1.62	2.29	0.7	1155	0.89
Patients	640	89.56 ±	37.48					
Controls	517	91.18 ±	40.32					
**Span forward**				3.17	2.97	1.06	1022	0.28
Patients	518	102.11 ±	19.56					
Controls	506	105.28 ±	64.83					
**Span backward**				−0.19	1.43	−0.13	1214	0.89
Patients	667	102.35 ±	24.91					
Controls	549	102.15 ±	24.80					
**Encryption**				2.8	1.85	1.51	971.193	0.13
Patients	522	104.63 ±	27.53					
Controls	486	107.43 ±	31.01					
**Short-term memory**				0.92	1.06	0.86	1180	0.38
Patients	621	103.18 ±	18.81					
Controls	561	104.1 ±	17.57					
**Delayed recall memory**				−4.3	2.50	−1.6	1658	0.09
Patients	885	117.49 ±	18.81					
Controls	852	113.19 ±	46.05					
**Recognition**				5.4	2.01	2.75	609	**0.006**
Patients	269	109.69 ±	21.42					
Controls	342	115.09 ±	27.03					
**Verbal fluency-letter**				1.12	1.98	0.56	1328	0.57
Patients	628	105.06 ±	36.76					
Controls	702	106.19 ±	35.49					
**Verbal fluency-animals**				1.62	1.98	1.07	1814	0.28
Patients	908	103.08 ±	31.15					
Controls	908	104.7 ±	33.24					
**Naming**				3.46	1.00	3.3	501.359	**0.001**
Patients	299	99.65 ±	15.52					
Controls	376	103.12 ±	10.49					

Cognitive performance in young and middle-aged adults (60 years old and above) vs. non-infected controls. Bold lettering indicates statistical significance.

Older adults were significantly impaired when compared to controls on Digit Span Forward performance (t = −4.52, *p* ≤ 0.001); delayed recall (t = 3.6, *p* < 0.001); recognition tasks (t = 6.94, *p* < 0.001); and the semantic verbal fluency task (t = 2.28, *p* = 0.02). In young and middle-aged adults, we found milder deficits in recognition and naming only (t = 2.75 and *p* = 0.006 and t = 3.3 and *p* = 0.001, respectively). Findings suggest mild attentional focus in young and middle-aged adults, whereas older adults display impairment in memory, language, and executive function.

### Severity of impairment: age-dependent phenotypes

To classify the severity of impairment, we used the deficit criterion as a difference of 1.5 standard deviations in performance on the tests administered. In young and middle-aged adults, we obtained four groups with distinct cognitive performance (Table 4):

- normal cognition (68.2%).
- impairment in one cognitive dimension (23.4%).
- impairment in two cognitive dimensions (6.5%).
- impairment in three cognitive dimensions impaired (1.8%).

**Table 4:** Frequency of cognitive impairment in all cohorts with (patients) and without infection (controls).

	**Controls**	**Patients**	**Total**
No impairment	1,032	1,381	2,413
Mild impairment (1 cognitive dimension affected)	306	523	829
Moderate impairment (2 cognitive dimensions affected)	75	156	231
Severe impairment (3 cognitive dimensions affected)	13	51	64

By strong contrast, older adults showed a distribution skewed toward more severe impairment:

- normal cognition (68.2%).
- impairment in one cognitive dimension (30.1%).
- impairment in two cognitive dimensions (11.3%).
- impairment in three cognitive dimensions impaired (3.7%).

The odds ratio (OR) of moderate to severe cognitive impairment (2 or 3 cognitive dimensions affected) was calculated for the entire sample (OR = 1.75), for young and middle-aged adults (OR = 0.71), and older adults (OR = 1.49). Thus, the risk was doubled in older adults compared to middle-aged and young adults.

### Predictors of risk of moderately severe or severe impairment

Moderately severe or severe impairment (2 or 3 dimensions) was significantly associated with infection diagnoses (chi-square = 26.57, *p* ≤ 0.0001) and with the presence and severity of anosmia (chi-square = 31.81, *p* ≤ 0.0001). No association was found between gender and cognitive dysfunction (chi-square = 0.74; *p* = 0.38) and between gender and anosmia (chi-square = 2.37; *p* = 0.12). The distribution of subjects by country is shown in Table 1, along with the average age and years of formal education; neither of these variables contributed to risk. On the other hand, the risk of moderate to severe cognitive impairment was nearly doubled in the presence of anosmia compared with its absence (OR = 1.89) (Table 5). Lastly, we also examined OR for cognitive impairment according to the severity of COVID-19 during the acute illness, considering the need for hospitalization, oxygen therapy, and admission to the intensive care unit. The risk of cognitive decline doubled when patients were hospitalized (OR = 2.06) or required intensive care (OR = 2.01). If they required oxygen therapy, the odds ratio was 1.47 (Table 6). Confidence intervals for each odds ratio are reported in Tables 5, 6, along with a normal deviation (z-statistic) calculated as ln(OR)/SE {ln(OR)} and the corresponding *p*-value.

**Table 5:** Risk of severe cognitive impairment with moderate vs. severe anosmia.

	**Anosmia**	
	**Severe**	**Moderate**
No impairment	525	720
Mild impairment	145	244
Moderate impairment	38	117
Severe impairment	9	47
Chi-Square =	30,81 (df = 3; p-value = 0.000)	
Odds ratio for severe impairment=	1.89 (95% CI: 1.35-2.64)	
z statistic =	3.766; p-value = 000002	

Includes cohorts from Argentina, Greece, Italy, and Russia.

**Table 6:** Risk of severe cognitive impairment in young vs. older adults (top) and stratified by severity of acute COVID-19 illness.

	**OR**	**95% CI**	**Z statistics**	***p*-value**
**Cognitive impairment vs. age**				
All sample	1.7578	1.35-2.28	4.218	0.0001
Older adults	1.4923	1.07-2.08	2.374	0.0176
Young and middle-aged adults	0.7191	0.42-1.24	1.192	0.2334
**Cognitive impairment vs. severity of COVID-19**				
Oxygen therapy (*N* = 154)	1.4696	0.97-2.22	1.832	0.07
Hospitalization (*N* = 81)	2.0653	1.44-2.96	3.952	0.0001
Intensive care unit (*N* = 9)	2.0065	0.91-4.39	1.738	0.08

## Discussion

We studied multiple samples of adults exposed to SARS-CoV-2 infection and collected them in a variety of settings (community as well as polyclinics or hospitals) in developed and underdeveloped countries, including individuals from multiple under-represented ancestries. Our analysis confirms that cognitive deficits are frequently present, but with different phenotypes determined by age at exposure and severity of anosmia. Older age and anosmia were found to be risk factors for severe phenotypes of cognitive impairment consistent with dementia-like syndrome. On the other hand, meta-analysis identified impaired verbal fluency in cases vs. controls independent of age. Notably, we did not detect inter-sample differences, which could be attributed to ancestry effects.

Attentional skills and executive functioning were significantly impaired in younger patients (59 and younger) across cohorts, but deficits were both infrequent and mild. A systematic literature review and retrospective meta-analysis on slightly < 1,000 mostly European patients revealed that following COVID-19, patients suffer from impaired memory, attention, and executive function verbal and specifically verbal fluency, regardless of acute disease severity (Daroische et al., 2021), but nearly half of the assessments were carried out by phone, reducing accuracy. More consistent with our findings, a community sample of young and middle-aged (< 54 years old) from Canada identified significant impairments in executive function (measured and reported) associated with acute infection severity (Hall et al., 2022), and identical results were reported in a smaller sample in Russia (Manukyan et al., 2022), and the USA (Apple et al., 2022). Of note, mild subjective impairment in younger adults is disproportionate to objective findings on formal testing and is associated with pre-existing psychiatric complaints (Apple et al., 2022).

Older adults were at over twice the risk of suffering memory, language, and executive function impairment that would be indistinguishable from early Alzheimer's disease with mild to moderate dementia. Once again, this finding is consistent with multiple previous reports in the literature (de Erausquin et al., 2021; Galderisi et al., 2024; Li et al., 2022; Tang et al., 2023; Gonzalez-Aleman et al., 2022; Miners et al., 2020) and many others. Similarly, as in most of the published literature, anosmia was significantly associated with cognitive impairment (Gonzalez-Aleman et al., 2022; Global Burden of Disease Long COVID Collaborators et al., 2022; Llana et al., 2023, 2022; Sohrabi et al., 2012; Yahiaoui-Doktor et al., 2019) and specifically with memory loss in recovering COVID-19 patients (Gonzalez-Aleman et al., 2022; Llana et al., 2022; Sohrabi et al., 2012). The prevalence of dementia-like cognitive impairment in these cohorts strongly contrasts with the published prevalence of only 5% Alzheimer's type dementia in people aged 65 to 74 (Alzheimer's Association, 2024). Regardless of the presumed mechanism of PASC-related cognitive impairment, with current reported rates of infection worldwide, a sharp increase in dementia-like syndromes would place a massive strain on health systems already burdened by this problem in aging populations (Alzheimer's Association, 2024).

The relationship between neurodegeneration and olfactory dysfunction is well established. For instance, poor olfactory discrimination has been shown to predict cognitive decline in older community-dwelling adults (Sohrabi et al., 2012). In the population-based LIFE-Adult Study (n = 6,783), better olfactory function was associated with better performance on cognitive tests even after adjustment for known confounders (Yahiaoui-Doktor et al., 2019).

Older age, anosmia, and cognitive decline are correlated with Alzheimer's disease and related dementia (ADRD). Entorhinal cortex thinning and anosmia are early hallmarks of memory decline (Murphy, 2019). Indeed, memory function and olfaction are intrinsically connected via neuroanatomical and functional pathways, leading to the hypothesis that exposure of the olfactory network to SARS-CoV-2 infection could account for tandem anosmia and cognitive impairment in at least a subset of COVID-19 and produce ADRD-like neuropathology (de Erausquin et al., 2021; Zamponi et al., 2021; Gonzalez-Aleman et al., 2022; de Erausquin et al., 2022). The current findings confirm the interconnection of anosmia and cognitive impairment following SARS-CoV-2 infection in a multinational sample.

Several independent studies have also provided support for the neuropathological and functional substrate of this hypothesis. In mammalian animal models, the olfactory networks have been identified as a potential route of neuroinvasion for SARS-CoV-2, which was shown to infect both brain endothelial cells and neurons in rhesus monkey (Jiao et al., 2021) and nasal epithelial, olfactory sensory neurons, and hippocampal dendritic spines in rodents (Kishimoto-Urata et al., 2022). In humans, a post-mortem study of COVID-19 patients identified viral particles in the olfactory neurons and their projections (Thakur et al., 2021). These findings were associated with microgliosis and elevated neuroinflammatory markers in cerebrospinal fluid Thakur et al., 2021, and both findings have been independently confirmed (Boutajangout et al., 2021; Poloni et al., 2021). An extensive review of neuropathological findings in 184 patients who died from complications of acute COVID-19 found microglial activation in close to half and detectable virus in the human cerebrum, cerebellum, cranial nerves, olfactory bulb, as well as in the olfactory epithelium (Lou et al., 2021).

Whether viral particles can persist in surviving patients remains an open and much more complex question to address. As suggested above, inflammatory processes independent of neuroinvasion may mediate an alternate mechanism. Analysis of frontal lobe transcriptomes donated by COVID-19 patients revealed that exposure to SARS-CoV-2 was associated with molecular signatures of aging mediated by type I/III interferon (Mavrikaki et al., 2022). The same innate immune pathway is associated with Alzheimer's disease pathobiology and immune-mediated neuronal injury in rodents (Roy et al., 2022). More directly relevant to this discussion, post-mortem brain tissue donated by COVID-19 patients revealed abnormalities in shared pathways associated with Alzheimer's disease, including those involved innate immunity, tau hyperphosphorylation, as well as a site-specific calbindin hypoactivity in the hippocampus (Reiken et al., 2022). Lastly, interleukins IL-1β, IL-6, and tumor necrosis factor plasma levels are elevated in PASC sufferers (Schulthei et al., 2022), and are also correlated to cognitive impairment in Alzheimer's disease (Silva et al., 2021), and with severity of hyposmia after COVID-19 (Liang et al., 2022).

Stimulation of the peripheral immune system by a disease-associated molecular pattern (DAMP) has been shown to result in an upregulation of complement-related genes in the hippocampus, even in the case of sterile DAMPs (Michalovicz et al., 2015). In the context of COVID-19, induction of type I interferon signaling and proinflammatory cytokines may affect brain function via vascular pathways; alternatively, direct infection of the olfactory neuroepithelium may alter brain function via molecular mimicry or inflammatory events through its projections to areas in the extended olfactory network. These events and the molecules involved may trigger neurodegenerative diseases such as neuropathology (Vavougios et al., 2022). Aside from molecules that can mediate these events directly and, in a dose-response manner (e.g., IL-6), adult neurogenesis sites such as the hippocampi and the olfactory network would be particularly vulnerable, accounting for the selective vulnerability of these networks and the resulting anosmia-cognitive impairment phenotype we report on. Of course, our results do not exclude other possible interpretations, such as vascular lesions or dysfunction of the olfactory pathway.

The results of our study should be interpreted in the context of its strengths and potential limitations. The main strength of our study is the use of prospective, harmonized data from a global collaboration of scientists working in different settings and with many underrepresented populations. The pooled analysis of individual-level data provides a unique insight into the phenotypes and clinical associations of cognitive impairment following COVID-19, which show several consistent aspects across centers. This was possible due to the inclusion of long-running cohorts functioning in the real-world setting initiated at the beginning of the pandemic. Another significant strength is that in most published studies, cognitive dysfunction may affect hospitalized patients in general, aside from diagnosis, whereas our cohorts include largely community-based samples with only an accurately proportional number of hospitalized cases.

Conversely, the most relevant limitation of our study is that this is a pooled analysis of heterogeneous studies rather than a multicentre study. Our approach's merit lies in assessing what amounts to real-world data and different approaches in diverse healthcare settings. For example, the population in Thessaly, Greece, represents an admixture of rural and urban areas and a small island complex. Similarly, data from the Canadian site is multi-ethnic as they serve a diverse community in an urban center, and the data from Argentina describe an Amerindian population rarely included in multinational studies. The heterogeneity of variables included in the analysis per study is another potential limitation. Studies included varying degrees of clinical, demographic, anthropometric, neuropsychological, fitness, and respiratory function assessments. Covariate analyses have accounted for these differences as far as reasonable.

We found distinct age-related phenotypes of cognitive impairment in patients recovering from COVID-19. Premorbid complaints emerged as the major predictor of mild impairment in young and middle-aged adults. In contrast, the severity of acute illness and the presence of olfactory dysfunction were the primary predictors of dementia-like impairment in older adults.

## Data Availability

The raw data supporting the conclusions of this article will be made available by the authors, without undue reservation.
